# Association Between Obsessive-Compulsive Symptom Dimensions in Mothers and Psychopathology in Their Children

**DOI:** 10.3389/fpsyt.2021.674261

**Published:** 2021-06-28

**Authors:** Thiago Blanco-Vieira, Marcelo Queiroz Hoexter, Marcelo C. Batistuzzo, Pedro Alvarenga, Natalia Szejko, Afonso Mazine Tiago Fumo, Eurípedes C. Miguel, Maria Conceição do Rosário

**Affiliations:** ^1^Child and Adolescent Psychiatry Unit (UPIA), Department of Psychiatry, Federal University of São Paulo, São Paulo, Brazil; ^2^Department of Psychiatry, Federal University of São Paulo, São Paulo, Brazil; ^3^Institute of Psychiatry, University of São Paulo, São Paulo, Brazil; ^4^National Institute of Developmental Psychiatry for Children and Adolescents (INCT-CNPq), São Paulo, Brazil; ^5^Department of Methods and Techniques in Psychology, Pontifical Catholic University, São Paulo, Brazil; ^6^Sírio-Libanês Hospital, São Paulo, Brazil; ^7^Department of Neurology, Yale School of Medicine, New Haven, CT, United States; ^8^Department of Neurology, Medical University of Warsaw, Warsaw, Poland; ^9^Department of Bioethics, Medical University of Warsaw, Warsaw, Poland; ^10^Hospital Central da Beira, Beira, Mozambique

**Keywords:** obsessive-compulsive symptoms, symptom dimensions, comorbidities, psychopathology, school age children, mother-child dyads

## Abstract

**Background:** The non-clinical presentation of obsessive–compulsive symptoms (OCS) in women may impact not only their daily lives and well-being but also increase the risk for emotional and behavioral problems in their children. This study aims to investigate the OCS dimension distribution in a large sample of mothers from a cohort of school age children and the association between these OCS dimensions with their own psychopathology, and with the presence of OCS and other psychopathology in their children.

**Method:** Our final sample consisted of 2,511 mother-children dyads recruited from the elementary schools of two large cities. Throughout multiple regression analysis, we examined the correlations between demographic and clinical variables of mothers assessed by the Mini International Psychiatric Interview (MINI) and the Dimensional Yale-Brown Obsessive-Compulsive Scale-Short Version (DY-BOCS-SV) with children's psychopathology status reported by the Child Behavior Checklist (CBCL).

**Results:** The overall prevalence of mothers who reported experiencing at least one OCS was 40% (*N* = 1,004). “Aggression/violence” was the most frequent symptom dimension (32.2%), followed by the “symmetry/ordering” (16.4%) and the “sexual/religious” dimensions (13.8%). There was a significant correlation between the presence of OCS and maternal psychopathology in general (*p* < 0.001, *r* = 0.397). Not only the presence but also the severity of the mother's OCS were strongly correlated to the total (*p* < 0.001), internalizing (*p* < 0.001), externalizing (*p* < 0.001), and OCS subscale scores (*p* < 0.001) on the CBCL.

**Conclusion:** OCS dimensions are highly prevalent in women. Presence and severity of maternal OCS are related to children's psychopathology and behavioral problems.

## Introduction

The lifetime prevalence of obsessive–compulsive disorder (OCD) in the general population is estimated to be around 1–2% (1). However, obsessive–compulsive symptoms (OCS) are much more prevalent than the full-blown OCD, ranging from 21 to 25% in the community (2) to more than 80% in clinical samples (3).

It is well-established that OCD may cause a lot of distress and interference not only to the patient but to the entire family (4–6). More recently, some studies have shown that non-clinical presentations of OCD may also cause a huge impact on family, social and academic functioning (7, 8). The presence of OCS has also been associated with an increased risk for specific psychiatric disorders in adults such as anxiety, mood and eating disorders (9). Particularly among women, since they are most often the children's main caregiver, the presence of OCS may increase the risk for OCD and/or other psychiatric disorders in their children, having a direct impact on the well-being of their offspring (10, 11).

For instance, Frías et al. (10) have found higher frequencies of the overprotective parenting style, among mothers with OCD, when compared with healthy controls. According to the authors, this dysfunctional parental style may partly account for higher levels of depression and anxiety in their children. Similarly, Coppola et al. (11) described that the presence of OCS in mothers from a community sample was associated with higher levels of parental stress and the presence of OCS in children. Other studies have shown that dysfunctional parenting, including overprotection and controlling, authoritarian and negative behaviors are more frequent in parents with OCS (12–14) and that they increase the risk for children to have higher levels of over responsibility, obsessional beliefs related to responsibility and threat estimation, as well as higher rates of psychiatric symptoms, including OCS (15).

Even though OCD was considered as a unique disorder for many years, more recent studies have demonstrated that OCD is a clinically heterogeneous condition and that the complex clinical OCD presentation can be summarized by a few symptom dimensions (or factors), such as the “contamination/washing,” the “symmetry/ordering,” the “hoarding,” the “aggressive/checking,” and the “religious/sexual/checking” dimensions (16–18).

These dimensions can be understood as a spectrum of potentially overlapping syndromes that may coexist in any patient and that extend beyond the traditional nosological boundaries of OCD. Furthermore, the dimensional approach addresses the OCD heterogeneity in light of a continuum of symptom severity, with persons without any OCS in one side of the continuum and very severe OCD patients on the other side of the same continuum (19). This approach includes symptoms ranging from a “no symptom at all” to a “most severe symptom” presentation, representing a more comprehensive assessment approach (20), particularly for community samples in which the subjects may OCS that do not fulfill the full-blown OCD presentation (16, 18).

Even though these OCS dimensions have been consistently replicated across studies, some studies suggested that the “aggressive/checking” and the “sexual/religious” form a unique factor (21–24), while others suggested that they should be broken down into two separate dimensions (23, 25–28).

These OCS dimensions have proven to be temporally stable, and associated with specific neuroimaging (29) and genetic findings (28, 30) as well as to treatment response (31).

It is now believed that this dimensional approach to phenotypic traits has the potential to advance our understanding of OCD and may aid in the identification of more robust endophenotypes (16). Therefore, identifying the distribution of OCS dimensions in the community (particularly in mothers) may be helpful for the early detection of OCD and for the development and implementation of treatment strategies, both for the patients and their families.

Notwithstanding, the distribution of OCS in a community sample of mothers has not been previously investigated. Furthermore, there is a need to investigate the association between OCS dimensions in mothers and psychiatric symptoms in their children. Altogether, these findings may add to the recognition of at-risk families for OCD, as well as to help in the development of prevention interventions for mothers and for their children (32).

Therefore, the present study aims to investigate the OCS dimensions distribution in mothers from a community sample of children aged 6–12 years. Additionally, we analyzed the associations between the mothers' OCS with other psychiatric symptoms as well as the associations between the OCS dimensions in mothers with the presence of OCS and other psychopathological symptoms in their children. We hypothesized that the mothers would have high frequencies of OCS, and that having OCS would increase their risk for other psychiatric disorders. We also expected positive correlations between the mother's OCS dimension severity and the severity of OCS and general psychopathology in their children.

## Materials and Methods

This study is part of a large Brazilian community-based cohort study known as the High-Risk Cohort (HRC) of the National Institute of Developmental Psychiatry, the INPD (inpd.org.br). A detailed description of the rationale, design, methods and preliminary results of the HRC can be found elsewhere (33).

**Figure 1:**
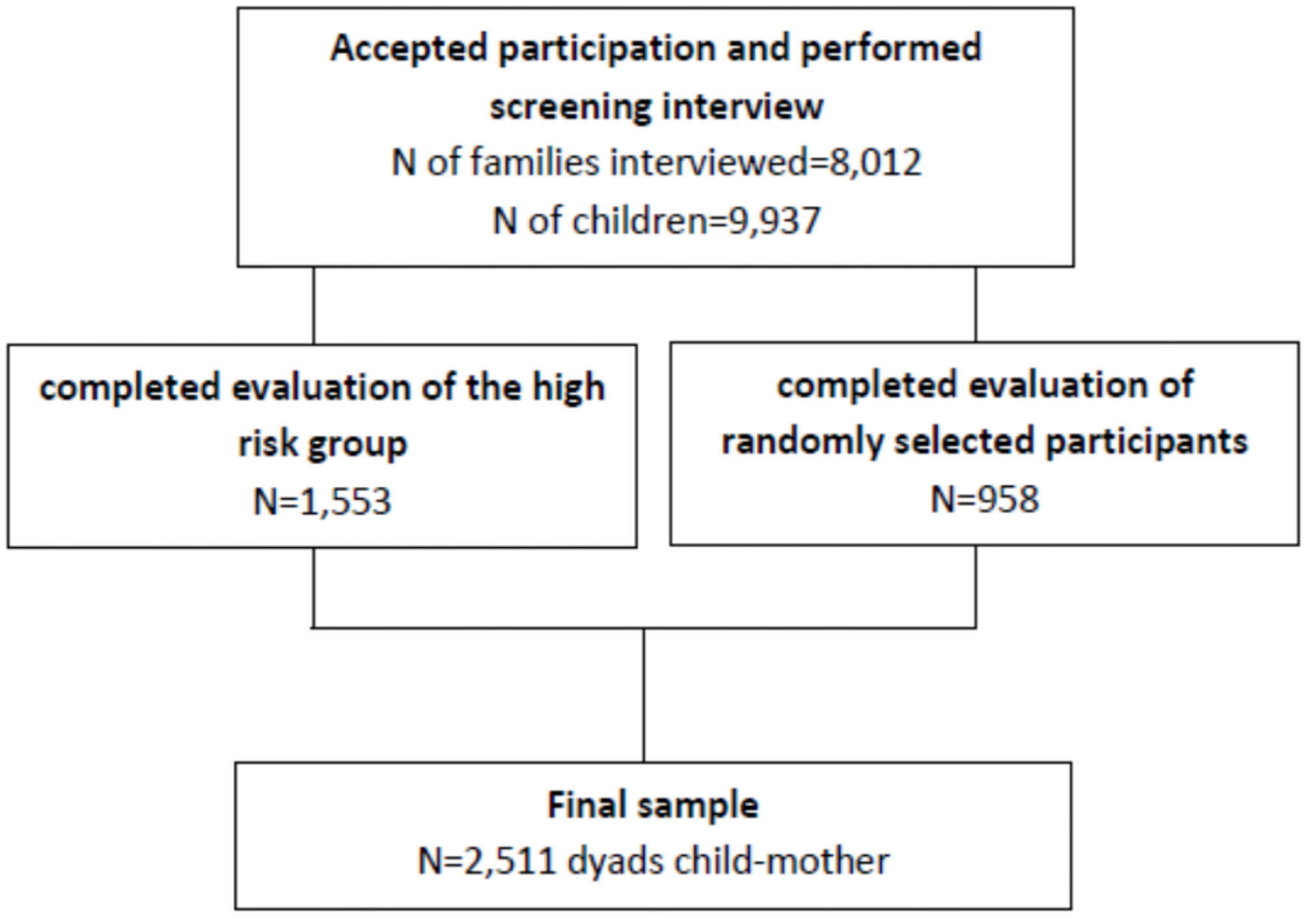
Sample selection process.

Briefly, families from a total of 57 public elementary schools in two large Brazilian cities (22 schools in Porto Alegre and 35 schools in São Paulo), were invited to participate in the study. All the interviews were conducted by trained lay interviewers with the main caregivers of children ages 6 to 12 years old (33). Figure 1 summarizes the sample selection process.

The study was approved by the Internal Review Boards (IRB) from both sites. After a thorough description of the study and the assurance that their decision to participate in the study would not interfere with their access to the schools, the main caregivers signed informed consents.

### Participants

Participants were 2,511 mothers and their children from a large community school-based cohort from the INPD (Figure 1).

### Instruments and Procedures

#### Questionnaire

Sociodemographic data was collected by a specific self-report questionnaire. Socioeconomic status was stratified according to the Brazilian Economic Classification Criteria instrument (ABA-ABIPEME) which defines the socioeconomic level of the individuals in five categories (from “A” to “E,” considering “A” as the highest and “E” as the lowest socioeconomic levels) based on a questionnaire that assesses number of household items owned by the families (i.e., refrigerators, washing machines, etc.) and the family's main provider educational level (34).

#### Maternal Assessment

Maternal OCS dimensions were ascertained by the Dimensional Yale-Brown Obsessive-Compulsive Scale-Short Version (DYBOCS-SV), developed for the screening of the presence and severity of OCS dimensions. All the items from the DYBOCS-SV were extracted from the full version of the DY-BOCS (20). Five OCS dimensions were assessed by 12 items briefly described below:

- “Aggression/violence” dimension: (1) “Do you have obsessions that something terrible (violent or aggressive content) is about to happen to yourself or to a relative close to you?; do you have worries that you may be responsible for this terrible event?; do you have violent or horrific images in your mind that something bad is about to happen?”; (2) “Do you need to check or take other measures to prevent or avoid harm coming to yourself and/or to others?; do you to avoid places or objects to prevent that something bad might happen to you or to others?”;
- “Sexual/religious” dimension: (3) “Do you have obsessions about sacrilege and/or blasphemy?; do you need to check to make sure that you have not done anything wrong of a religious nature”; (4) “Do you have to repeat an action over and over again after having a religious obsessional thought?; do you need to check or avoid something to prevent terrible consequences from having religious obsessions?”; (5) “Do you have forbidden or improper sexual thoughts, images or impulses?; do you have obsessions about violent sexual behavior toward other people”; (6) “Do you have to avoid certain actions, people, places or things or do you have to repeat an action over and over again in order to prevent sexual obsessions from occurring?; do you have to check to make sure that you have not done anything wrong of a sexual nature”;
- “Symmetry/ordering” dimension: (7) “Do you have obsessions about things needing to be perfect or exact or “just-right”?; “do you have obsessions about symmetry?; “(8) “Do you have ordering and/or arranging compulsions?; do you have counting compulsions?; do you have compulsions that involve symmetrical touching of objects or people and/or evening-up behaviors?”; do you have to avoid certain actions, people, places or things to prevent obsessions about symmetry or exactness from occurring”?;
- “Contamination/cleaning” dimension: (9) “Are you obsessed with dirt or germs?; are you overly concerned or disgusted with body waste or secretions?; are you bothered by sticky substances or residues?,” (10) “Do you have compulsive or ritualized hand washing, showering, bathing or toilet routines?; do you have compulsions (or rituals) that involve repeated cleaning of households items or other inanimate objects?; do you have to do something to prevent or remove contact with contaminants? Do you avoid certain places because of contamination concerns?”;
- “Collecting/hoarding” dimension: (11) “Do you have obsessions about needing to save or hoard things for the future?; do you have obsessions about losing things?”; (12) “Do you have compulsions to hoard or collect things?; do you avoid certain actions, people, places or things to prevent from having to collect something?.”

Each of these items were assessed on a 0 to 5 severity scale (0 = no symptoms, 5 = severe symptoms), yielding a total dimension score ranging from 0 to 10. For this study we combined the scores from the “sexual/religious” dimension which ranged from 0 to 20. The DYBOCS-SV total score may vary between 0 and 60. Since we used a brief version of the DY-BOCS, the internal consistency was calculated using the Cronbach's alpha.

The maternal history of psychiatric disorders was assessed using the Mini International Psychiatric Interview (MINI) (35) and the MINI Plus (36) based on DSM-IV criteria. The following modules were used: (1) bipolar disorder; (2) mood disorders; (3) panic disorder; (4) anxiety disorders; (5) drug abuse and dependence; (6) psychotic disorders; and (7) attention deficit hyperactivity disorder (ADHD). The overall MINI Plus inter-rater reliability is satisfactory for the diagnostic categories (kappa coefficient ranging from 0.86 to 1) (37).

#### Children's Assessment

The Child Behavior Checklist (CBCL) was used to assess the presence and severity of Obsessive-Compulsive Symptoms as well as the overall psychopathology. The CBCL (38) is one of the most widely used instrument to assess behavioral problems in children. It was translated to Portuguese and validated in Brazil by Bordin et al. (39). The CBCL is composed of 113 questions, scored on a three-point Likert scale (0 = absent, 1 = occurs sometimes, 2 = occurs often). The CBCL provides 3 main scores: internalizing, externalizing a total psychopathology severity rating. The original version of the CBCL has good test-retest reliability (0.90) and internal consistency (Cronbach's alpha ranging from 0.72 to 0.97) (39).

The presence and severity of OCS in children was quantified using the CBCL-OCS subscale proposed by Nelson et al. (40), which consists of eight items from the CBCL, with scores varying from 0 to 16. The CBCL-OCS subscale is composed of the following CBCL items: (9) can't get his/her mind off certain thoughts, obsessions; (31) feels might think or do something bad; (32) feels he/she has to be perfect; (52) feel too guilty; (66) repeats certain actions over and over, compulsions; (84) engages in strange behavior; (85) has strange ideas; (112) worries. This CBCL-OCS subscale has demonstrated good reliability and validity in discriminating children and adolescents with OCD (41). The Cronbach's alpha for the 8 items was 0.87 and the factor loading had positive values ranging from 0.514 to 0.769 (41).

### Data Analysis

The internal consistency between the DYBOCS-SV items was calculated using the Cronbach's alpha.

All the data (including the sociodemographic data, the maternal psychiatric conditions, the OCS dimensions and severity scores and the children's CBCL scores) were analyzed using the Statistical Package for Social Sciences (SPSS) version 20.0. All statistical tests were 2-sided using a significance level set at *p* < 0.05.

The variables were tested for normality using the Kolmogorov-Smirnov test. Since all variables showed non-parametric distributions, the comparison of means between two or more groups was run using the Mann-Whitney and Kruskal-Wallis tests, respectively. The pairwise comparisons were corrected with Dunn-Bonferroni method (42), if required, considering an overall significance level of 0.05. The correlations between the continuous variables (maternal OCS dimensions severity scores and the children's psychopathology CBCL scores) were analyzed with the Spearman test.

To evaluate the effects of the maternal characteristics (explanatory variables) on the CBCL symptom dimensions (dependent variables) we used univariate and multivariate linear regressions. The variable selection method for the regression modeling was the backward variable elimination. Following this procedure, all of the variables were entered initially in the model in a single step and then the variables were removed one at a time if the level of significance given by *p* <5%. The order of elimination followed the poorer result across the remaining group of variables.

Considering that the Ordinary Least Squares (OLS) method states that the sampling distribution of the coefficients approximates a normal distribution as the sample size becomes larger, we decided to use the a linear regression model even though the variables showed a non-parametric distribution (43).

The multivariate linear regression models were built including all demographic and maternal psychopathological variables as the explanatory variables and the children's CBCL psychopathological domains as the outcome variables (44, 45).

Additionally, in order to predict the risks for the children's psychopathology according to the mothers' severity of the OCS we built a decision tree. The decision trees provide a framework to quantify the values of outcomes and the probabilities of achieving them, then representing a valuable tool to reveal cutoff points to predict the risks within conditions in analysis (46).

## Results

The mothers comprised a group of women with ages ranging from 20.4 to 58.1 (mean = 36.4, SD = 6.9). The majority of participants self-declared as being white (57.8%, *N* = 1,452). Approximately 42.7% were classified as part of the A/B income class. The majority of the sample (67.5%) was married or had a partner. Regarding the educational level, 72.1% had a college degree, approximately 3.4% had finished high school and 24.4% did not complete elementary school. Among the children, the mean age was 10.2 (SD = 1.9) and 45.2% were female (*N* = 1,136).

The overall prevalence of mothers who reported experiencing at least one OCS was 40.0% (*N* = 1,004). “Aggression/violence” was the most frequent symptom dimension (32.2%), followed by “symmetry/ordering” (16.4%), “sexual/religious” (13.8%), “contamination/cleaning” (11.2%), and “collecting/hoarding” (10.9%) (Table 1).

**Table 1:** Maternal sociodemographic and clinical characteristics.

	***N* = 2,511**
**Age (mean** **±** **SD) years**	36.4 ± 6.9
**Marital status**, ***N*** **(%)**	
Without partner	807 (32.5)
With partner	1,678 (67.5)
**Educational level**, ***N*** **(%)**	
Illiterate/incomplete elementary	607 (24.4)
Complete elementary/ incomplete middle	1,791 (72.1)
Finished College and Higher	85 (3.4)
**Socioeconomic status**, ***N*** **(%)**	
A/B	878 (42.7)
C	1,017 (49.5)
D/E	160 (7.8)
**Psychiatric disorders**, ***N*** **(%)**	
Bipolar disorder *lifetime*	166 (6.6)
Any mood disorder *current*	491 (19.6)
Panic disorder *lifetime*	216 (8.6)
Any anxiety *current*	588 (23.4)
Any substance use related disorder *current*	33 (1.3)
Psychotic syndrome *lifetime*	170 (6.8)
Attention deficit hyperactivity disorder *lifetime*	44 (1.8)
**Any OCS symptom**, ***N*** **(%)**	1.004 (40.0)
Aggression/violence	809 (32.2)
Sexual/religious	347 (13.8)
Symmetry/ordering	411 (16.4)
Contamination/cleaning	281 (11.2)
Collecting/Hoarding	273 (10.9)
**DYBOCS-SV scores, mean** **±** **SD**	
Global score	3.6 ± 6.9
Aggression/violence score	1.4 ± 2.5
Sexual/religious score	0.6 ± 2.0
Symmetry/ordering score	0.7 ± 1.9
Contamination/cleaning score	0.5 ± 1.5
Collecting/Hoarding score	0.4 ± 1.3

The Cronbach's alpha coefficient for the DYBOCS-SV showed good internal consistency for all DYBOCS-SV domains: global score = 0.853; aggression/violence symptom dimension = 0.791; sexual/religious = 0.708; symmetry/ordering = 0.847; contamination/cleaning = 0.782; and collecting/hoarding = 0.656.

The most frequent psychiatric disorders in the mothers were anxiety disorders (23.4%, *N* = 588). Higher rates of comorbidity for almost all of the assessed DSM-IV psychiatric disorders were found in mothers with OCS as compared to subjects with no OCS (*p* < 0.001). Table 2 presents the sociodemographic and clinical characteristics of the children.

**Table 2:** Children's sociodemographic and clinical characteristics.

**Age** (mean ± SD) years	10.2 ± 1.9
**Gender–Female, *N* (%)**	1,136 (45.2)
**CBCL**	
Total score	26.9 ± 25.1
Internalizing Score	8.7 ± 8.7
Externalizing Score	8.5 ± 9.1
OCS subscale score	1.6 ± 2.2

Positive and statistically significant correlations were found between the severity of the maternal OCS and all of the CBCL domains (global, externalizing, internalizing, OCS). The higher correlation coefficient was found between maternal DY-BOCS-SV total score and children's CBCL total score about 0.382.

Multivariate linear regression analyses of the CBCL total, internalizing, externalizing and OCS scores according to maternal and children characteristics. These analyses pointed that not having a partner (being single, divorced or a widow), having any mood (p < 0.001) or anxiety disorder (*p* < 0.001), and reporting a history of ADHD (*p* = 0.005) during childhood increased the risk for children to have higher rates of externalizing, internalizing and total CBCL scores.

The presence and severity of maternal OCS also increased the risk for behavioral and emotional problems in children.

Among the OCS dimensions, the “aggression/violence” dimension significantly increased the risk for higher rates of all CBCL psychopathological domains (total, B = 1.03, 0.59–1.48, *p* < 0.001; externalizing, B = 0.35, 0.19–0.52, *p* < 0.001; and internalizing, B = 0.33, 0.17–0.48, *p* < 0.001; obsessive compulsive, B = 0.08, 0.04–0.12, *p* < 0.001). The “sexual/religious” OCS dimension was associated with the children's CBCL internalizing scores (B = 0.23, 0.05–0.40, *p* = 0.014) and to OCS in children (B = 0.09, 0.04–0.14, *p* < 0.001). The “symmetry/ordering” OCS dimension was associated to higher frequencies of total CBCL scores (B = 1.08, 0.47–1.68, *p* = 0.001), to externalizing (B = 0.47, 0.27–0.67, *p* < 0.001) and to internalizing (B = 0.23, 0.02–0.44, *p* = 0.03) CBCL scores in children. The contamination/cleaning dimension was associated to CBCL total score (B = 0.87, 0.14–1.61, *p* = 0.02), internalizing score (B = 0.35, 0.10–0.61, *p* = 0.007) and children OCS score (B = 0.14, 0.08–0.20, *p* < 0.001). Also, this was the maternal OCS dimension which showed higher correlation to children's OCS scores. Finally, mothers who report collecting/hoarding symptoms have more frequently children with behavioral and emotional overall symptoms (B = 1.19, 0.44–1.94, *p* = 0.002) and externalizing problems (B = 0.47, 0.19–0.76, *p* = 0.001) (Table 3).

**Table 3:** Multivariate linear regression analyzing CBCL internalizing, externalizing and OCS scores according to maternal and children's characteristics.

	**CBCL total**		**Internalizing symptoms**		**Externalizing symptoms**		**Obsessive compulsive symptoms**	
	**Adjusted coefficient (CI95%)**	***p***	**Adjusted coefficient (CI95%)**	***p***	**Adjusted coefficient (CI95%)**	***p***	**Adjusted coefficient (CI95%)%**	***p***
**Maternal characteristics**								
**Age** (years)	–	–	–	–	–	–	–	–
**Marital status**—ref. Without partner	3.63 (1.76–5.5)	<0.001	0.86 (0.22–1.51)	0.009	1.37 (0.66–2.07)	<0.001	–	–
**Educational level**—ref. Complete elementary		–				–		
Illiterate/incomplete elementary	–	–	–	–	–	–	–	–
Finished college and higher	–	–	–	–	–	–	–	–
**Socioeconomic status**—ref.=C income class		–				–		
A/B income class	–	–	–	–	–	–	–	–
D/E income class	–	–	–	–	–	–	–	–
**Maternal psychiatric condition**								
Bipolar disorder *lifetime*	–	–	–	–	–	–	0.47 (0.10–0.83)	0.012
Any mood disorder *current*	10.06 (7.39–12.74)	<0.001	3.63 (2.71–4.56)	<0.001	2.92 (1.91–3.93)	<0.001	0.47 (0.22–0.71)	<0.001
Panic disorder *lifetime*	–	–	–	–	–	–	–	–
Any anxiety *current*	10.21 (7.59–12.84)	<0.001	3.87 (2.96–4.78)	<0.001	2.59 (1.60–3.58)	<0.001	0.69 (0.46–0.92)	<0.001
Any substance use related disorder *current*	–	–	–	–	–	–	0.78 (0.08–1.49)	0.030
Psychotic syndrome *lifetime*	–	–	–	–	–	–	–	–
Attention deficit hyperactivity disorder *lifetime*	9.55 (2.92–16.19)	0.005	3.27 (0.98–5.57)	0.005	3.09 (0.58–5.59)	0.016	–	–
**DYBOCS–SV**								
Aggression/violence score	1.03 (0.59–1.48)	<0.001	0.33 (0.17–0.048)	<0.001	0.35 (0.19–0.52)	<0.001	0.08 (0.04–0.12)	<0.001
Sexual/religious score	–	–	0.23 (0.05–0.40)	0.014	–	–	0.09 (0.04–0.14)	<0.001
Symmetry/ordering score	1.08 (0.47–1.68)	0.001	0.23 (0.02–0.44)	0.030	0.47 (0.27–0.67)	<0.001	–	–
Contamination/cleaning score	0.87 (0.14–1.61)	0.020	0.35 (0.10–0.61)	0.007	–	–	0.14 (0.08–0.20)	<0.001
Colectting/hoarding score	1.19 (0.44–1.94)	0.002	–	–	0.47 (0.19–0.76)	0.001	–	–
**Children characteristics**								
**Age (years)**	–	–	0.26 (0.10–0.42)	0.002	–	–	–	–
**Gender** (ref. Male)	2.98 (1.23–4.73)	0.001	–	–	1.48 (0.82–2.15)	<0.001	–	–
*N*	2.485		2.485		2.485		2.511	
*R*^2^	21.9%		22.0%		15.1%		15.6%	
*R*^2^ adjusted	21.6%		21.7%		14.9%		15.4%	

**Figure 2:**
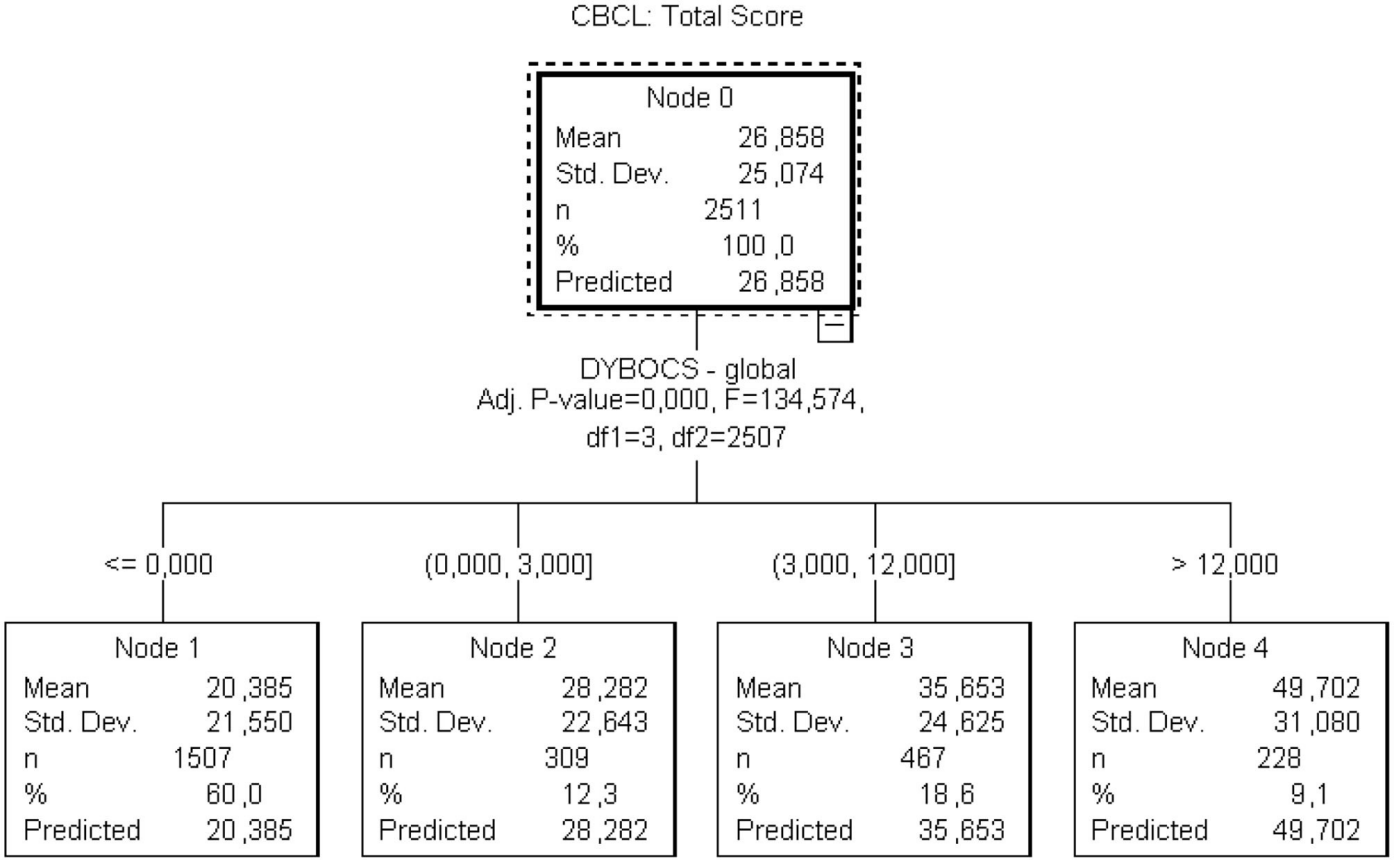
Decision tree regarding maternal OCS global score and children's overall psychopathology.

**Figure 3:**
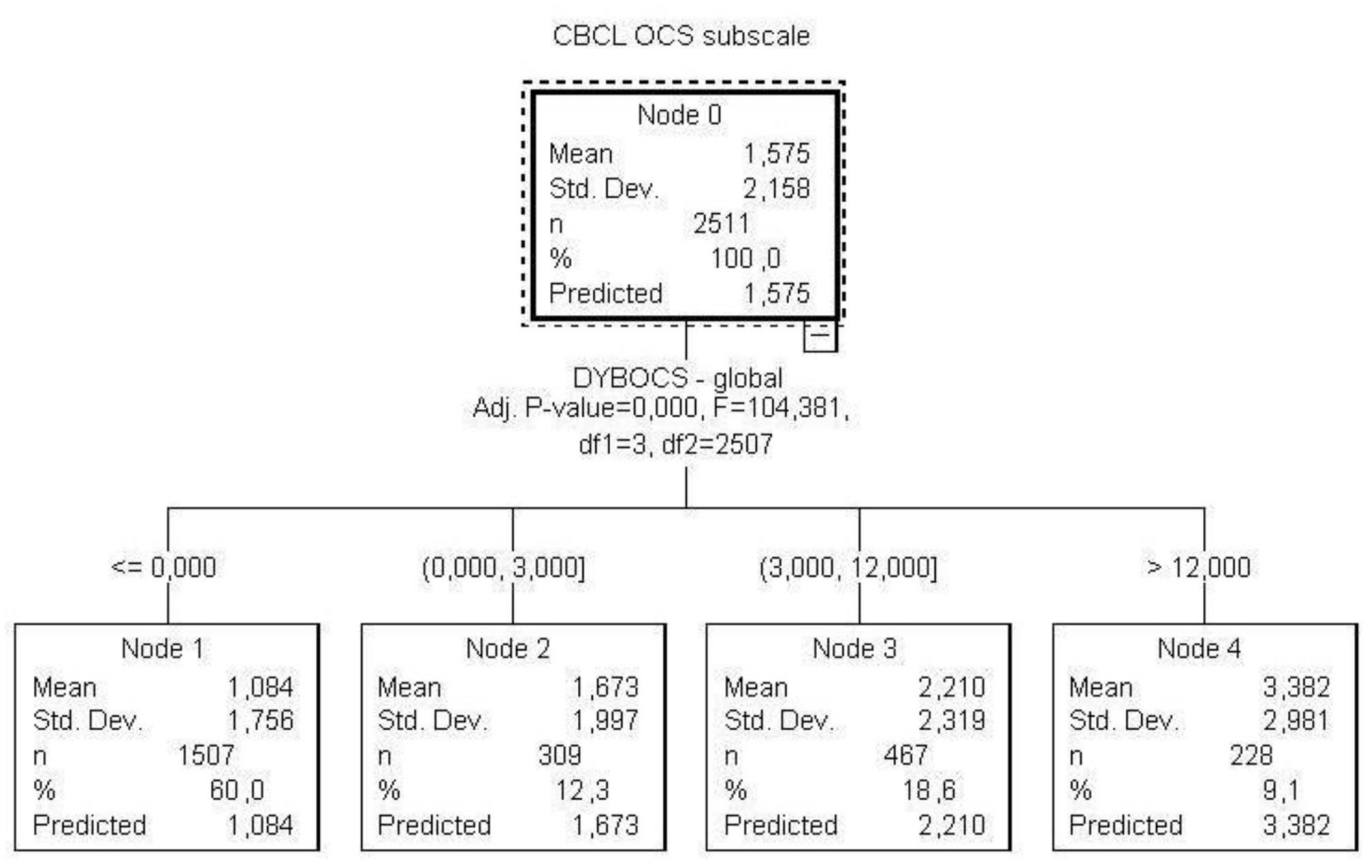
Decision regarding maternal OCS global score and children's OCS.

The decision three model showed that a maternal DYBOCS-SV score equal or higher than 12 was associated with approximately a 14 points higher scores on the CBCL total score and a 3 points higher on the OCS subscale (Figures 2, 3).

## Discussion

To our knowledge, this is one of the few reports of the prevalence of OCS dimensions in adult women and the association of maternal OCS with children's psychopathology in a large epidemiological sample. The analyses demonstrated a high prevalence rate of OCS in the mothers and that the presence of the OCS dimensions were associated with higher rates of DSM-IV psychiatric disorders in the mothers and with OCS and general psychopathology in their children.

There are few studies investigating the prevalence of OCS in community samples (2, 47–55). The current study showed that 40% of the women interviewed reported at least one OCS and that the “aggression/violence” and the “symmetry/ordering” OCS dimensions were the most frequent (reported by 32.2 and 16.4% of the women, respectively). These rates are in accordance to the rates reported by Alvarenga et al. (47), but they are higher than the rates reported in other studies (2, 48–55).

This difference between the results may be explained by methodological issues. For instance, in the current study, the women were directly interviewed by well-trained interviewers. Considering the secrecy characteristic of OCD, it is possible to hypothesize that subjects report their OCS more openly when are directly interviewed. Additionally, some studies have reported that in community samples, women tend to present higher frequencies and severity of OCS, when compared to men (2, 47, 51) and clinical samples (56–59).

The presence of OCS was associated with elevated rates of all other DSM-IV disorders assessed with the MINI. More specifically, anxiety, mood and psychotic disorders were strongly associated with the presence of OCS. It is well-established that OCD is strongly associated with high comorbidity rates with other psychiatric disorders (49, 52, 54, 60–71). The current study has shown that even without the full-blown OCD expression, the presence of OCS is also associated with higher risks for psychiatric disorders. These findings reinforce the idea that the screening of OCS is extremely important for the early identification and treatment of OCD as well as other psychiatric symptoms.

The current results have shown that the lifetime presence of maternal ADHD and the current presence of mood and anxiety disorders had an independent significant increase in the CBCL OCS, internalizing, externalizing and total scores in their children. There is a huge body of evidence that children of mothers with anxiety and/or depression symptoms have higher frequencies of emotional and behavioral problems (72, 73). Studies analyzing the emotional impact of parental OCD on their children have shown that these children are at higher risk of suffering from mental disorders in general (10) and having internalizing, but not externalizing symptoms (72–75). Our results expand these findings showing correlations between maternal OCS to all CBCL domains (internalizing, externalizing and OCS).

We have also assessed the impact of maternal OCS in their offspring. All OCS dimensions in the mothers were associated with higher rates of CBCL internalizing, externalizing, OCS and total scores in their offspring. Interestingly, each OCS dimension had specific associations with the CBCL domains. For instance, the aggression/violence dimension was significantly associated with all CBCL domains as well as to the total CBCL scores. On the other hand, the sexual/religious dimension was significantly associated only with the internalizing and the OCS domains. The symmetry/ordering dimension was associated with the overall, externalizing and internalizing CBCL domains, the contamination/cleaning dimension was associated with the overall, internalizing and OCS domains and the hoarding/collecting dimension was associated with the overall and externalizing CBCL domains. These results emphasize the idea that OCD is a heterogeneous disorder and that each OCS dimension has specific clinical correlates. Therefore, a dimensional approach may be used in future studies in order to reduce the negative impact of this heterogeneity on the interpretation of the study results.

Coppola et al. (11) have reported the results from a non-clinical sample of mothers with OCS in which the presence of maternal OCS were significantly related to OCS in the offspring and that this finding was mediated by parental stress. The authors hypothesized that OCS lead mothers to experience more parental stress or to display more dysfunctional (and less warmth/affection) parenting styles, what may increase the risk for childhood psychopathology (73, 75–77).

Our results suggest a familial aggregation of the OCS between mothers and their offspring. Previous studies have suggested that this may be due to environmental (73, 75–77) and/or genetic (78–80) effects. Considering that studying the heritability of the OCS was not an objective of the current study, we hope that future family, twin or genome scan studies may incorporate the assessment of OCS dimensions.

Additionally, the maternal marital status was independently associated with the expression of emotional and behavioral problems but not to OCS in their children. Mothers who did not have a partner at the time of the interview showed higher rates of externalizing, internalizing and total CBCL scores. Other studies have also demonstrated that single mothers have higher risks of emotional distress and disruptive parenting practices and in consequence their children are more vulnerable to behavioral and emotional problems (81).

The children's male gender was independently associated with higher risks for having externalizing and total CBCL higher scores. It is important to mentions that male gender has been pointed as an independent risk for behavioral problems in childhood (82, 83).

Our findings should be considered in light of several limitations. First, we did not control the effects of maternal OCS in children according to other important characteristics such as family environment, mother-child interaction quality, parental skills, and social support in general. Second, only the mothers were interviewed about themselves and their children. Future research should use multi-informant approaches. Third, the presence of other disorders may interfere in the impact of the mother's OCS on their children's psychopathology. Fourth, the internal consistency of the hoarding dimension was lower than the internal consistency of the other OCS dimensions. Finally, the current study focused solely on the role of maternal OCS. Fathers have been historically underrepresented in research on parent-child interactions and the inclusion of these analyses in future studies may reveal important knowledge to the field.

Despite these limitations, the current study has demonstrated that OCS dimensions are highly prevalent in women from community samples and that the presence and severity of OCS are associated with higher risks for them to have higher comorbidity rates. Furthermore, maternal OCS were associated with OCS and general psychopathology in their children and these associations varied according to specific OCS dimensions, reinforcing the relevance of using a dimensional approach to assessing OCD. Other characteristics such as not being married and having a current and/or lifetime history of ADHD, mood and anxiety disorders were also associated with higher CBCL scores.

## Conclusion

OCS dimensions are prevalent and are associated with comorbid psychiatric disorders in women. Presence and severity of maternal OCS are associated with OCS and general psychopathology in their offspring and these associations vary according to specific OCS dimensions. All together, these findings reinforce the relevance of screening for OCS and for the development and implementation of preventive strategies for adults with OCS and their children.

## Data Availability Statement

The raw data supporting the conclusions of this article will be made available by the authors upon request and analysis of the coordination of the National Institute of Developmental Psychiatry for children and adolescents (INCT-CNPq).

## Ethics Statement

The studies involving human participants were reviewed and approved by Research Ethics Committee of the University of São Paulo, Research Ethics Committee of the Federal University of Rio Grande do Sul. Written informed consent to participate in this study was provided by the participants' legal guardian/next of kin.

## Author Contributions

TB-V has participated on conceiving the idea of the project, in the data analyses, and article preparation. MH has participated in the research design and article preparation. MB, NS, and AF have participated in data analyses and article preparation. PA and EM have participated in the research design, data analyses, and article preparation. MR has participated in the research design, mentorship activities, data analyses, and article preparation. All the authors discussed the results and contributed to the final manuscript. After all, there was agreement on the final version which is now submitted to the editorial board appreciation.

## Conflict of Interest

The authors declare that the research was conducted in the absence of any commercial or financial relationships that could be construed as a potential conflict of interest.
